# Health Tract, No. 134

**Published:** 1865

**Authors:** 


### HEALTH TRACT, No. 134.

### POISONS AND ANTIDOTES.

THE antidote of a poison is that which renders it instantly harmless; this it does by converting the elements or ingredients of the poison into new compounds, which are wholly innocuous. But in all these cases, the benefits to be derived from the employment of an antidote, are proportioned to the instantaneousness of the application; the importance of this is very generally understood, but it serves to deprive friends of all presence of mind; they are thrown into such a flurry, as to be incapable of connected thought, or efficient action. It may therefore save many a human life, if the reader will impress upon his mind two or three general principles. It is true, that "every bane has its antidote," but as there are hundreds of poisons, and the memory would be overtaxed with an antidote for each, it is agreeable to note that some substances are perfect antidotes against a dozen poisons; and it is fortunate, too, that these substances are almost always at hand, even in the poorest households. Strong coffee; salt and mustard; white of eggs; any kind of domestic oil, lard or grease—these four things antagonize almost all ordinary poisons. If the reader will bear this in mind, he can be happily and efficiently calm, under almost any circumstances of poison, in which he is likely to be placed.

1. Prevention is best. No poisonous substance should be allowed in any household for one single instant, after it is out of the hand; whatever has been left after use, should be at once thrown into the sink, or carried out into the street or road, broken, poured out or scattered.

2. The very moment you see any thing in a paper or bottle or other vessel, without a mark showing what it is, empty it without a moment's delay into the sink; this is safer than throwing it into the fire, for it may be inflammable or explosive, and cause much mischief.

3. Never take, taste, or give any thing, whether powder or fluid in the dark, or without looking deliberately at the label, in a clear light, although you may have put the vessel or paper down with your own hand, a minute before.

But from inattention, recklessness, or design, poisons will sometimes be swallowed, and the truly wise will inform themselves beforehand, as to the best means of procedure.

1st. Send for a physician. Meanwhile, remember that the effect of administered poison is instantaneous, or comes on slowly. If instantaneous, the patient immediately cries out with the sensation of heat or burning, or scalding at any point from mouth to stomach; the presumption then is, that some corrosive poison has been taken; something which eats or destroys or disorganizes the muscles or fleshy parts of the tongue, mouth, throat, stomach, etc.; most poisonous substances of this sort are acids, and the first best remedy likely to be at hand, is common soap dissolved in water, or soda or saleratus or magnesia; but in the hurry of inexpert hands the remedy may be made so strong as to become of itself another poison, hence it is best to take the simplest thing which is most likely to be at hand, and which can not injure in any quantity or strength in which it can be taken; hence for poisons which cause an instantaneous sensation of burning in the throat, etc., drink a tea-cupful of sweet oil or lard or grease of any sort; the most that can happen from an over amount is that it will be vomited up, and this brings more or less of the poison out of the stomach; then you can more leisurely drink magnesia-water or strong soapsuds, or a table-spoon of wood-ashes, put in half a pint of lukewarm water, stir, let it settle two minutes, pour it off and drink.

If a powder has caused the urgent sensations, the most generally applicable antidote is to swallow one or two raw eggs; the white is the efficient part, but there may not be time to separate the yolk this is best in poisons from arsenic, corrosive sublimate, verdigris, creosote, etc.

If the effect is not instantaneous, and time may be taken, the first best thing to be done in all cases is to get the poison out of the stomach instantly, by swallowing every five minutes a tea-cup of warm water into which has been stirred a full tea-spoon each of common salt, and ground kitchen-mustard; there is vomiting almost as soon as it reaches the stomach; then drink a cup or two of very strong coffee, which is the best remedy for all anodyne poisons, as opium, morphine, laudanum, etc., etc. In short, if the sufferings are instantaneous and urgent, drink sweet oil or soapsuds; if gradual or causing drowsiness, mustard emetic, strong coffee or white of eggs.

